# First description of the female of *Sinopoda serrata* (Wang, 1990) (Araneae, Sparassidae)

**DOI:** 10.3897/zookeys.321.5752

**Published:** 2013-08-07

**Authors:** Dan Quan, Jian Chen, Jie Liu

**Affiliations:** 1 College of Life Sciences, Hubei University, Wuhan 430062, Hubei, China

**Keywords:** Taxonomy, biodiversity, systematics, huntsman spiders

## Abstract

The female of *Sinopoda serrata* (Wang, 1990) is described for the first time from Tiantangzhai National Forest Park, Hubei province, China. This species has been recorded from the region of Central China. Morphological descriptions and illustrations of this species are given.

## Introduction

The spider genus *Sinopoda* Jäger, 1999 is distributed in East Asia and northern parts of South East Asia with 49 species described so far, 32 of which are known from China (Jäger 2012, Platnick 2013).

The species *Sinopoda serrata* (Wang, 1990) was first described in *Heteropoda* Latreille, 1804, based on male specimens only from Mt. Lushan (Jiangxi Province, China) and Mt. Huangshan (Anhui Province, China) (Wang 1990). Jäger (1999) transferred this species to the genus *Sinopoda*. Recently, the authors examined specimens collected from Hubei Province and found that 2 females and 7 males seemed to be this species based on comparison with the type specimens. Based on the currently known specimens, this species is distributed in the region of Central China (Fig. 17). The aim of the current paper is to re-describe the male and report the female for the first time, providing detailed morphological illustrations and photos.

## Material and methods

Specimens were examined with an Olympus SZX16 stereomicroscope; details were further investigated with an Olympus BX51 compound microscope. All illustrations were made using an Olympus drawing tube. Male palp and epigyne were examined and illustrated after dissection from the spider bodies. Photos were made with a Canon G10 digital camera (14.7 megapixels) mounted on an Olympus SZX16 stereomicroscope. The digital images depicting the habitus and genital morphology were a composite of multiple images taken at different focal planes along the Z axis and assembled using the software package Helicon Focus 3.10. Most hairs and macrosetae were usually not depicted in the palp and epigyne drawings.

Leg measurements are shown as: total length (femur, patella, tibia, metatarsus, tarsus). Number of spines are listed for each segment in the following order: prolateral, dorsal, retrolateral, ventral (in femora and patellae ventral spines are absent and fourth digit is omitted in the spination formula).

Abbreviations: ALE — anterior lateral eyes, AME — anterior median eyes, C — conductor, E — embolus, EA — embolic apophysis, FD — fertilization duct, GA — glandular appendage, LF — lateral furrow, LL — lateral lobes, LS — lobal septum, MSu — membranous sac unexpanded, PLE — posterior lateral eyes, PME — posterior median eyes, PP — posterior part of spermathecae, RTA — retrolateral tibial apophysis, T — tegulum. I, II, III, IV — legs I to IV. Collections: HBU — Hubei University, Wuhan, China; HNU — Hunan Normal University, Changsha, China.

## Taxonomy

### Family Sparassidae Bertkau, 1872
Subfamily Heteropodinae Thorell, 1873
Genus *Sinopoda* Jäger, 1999

#### Sinopoda serrata

(Wang, 1990)

http://species-id.net/wiki/Sinopoda_serrata

Figs 1 –17

Heteropoda serrata Wang, 1990: 10, figs 17–19 (description of male).
Sinopoda serrata Jäger, 1999: 21 (transferred from *Heteropoda*).
Sinopoda serrata *Sinopoda serrata* Song et al., 1999: 469, fig. 270O (illustration of male).

##### Type material examined.

1 ♂ (holotype, HNU), Mt. Lushan, Jiangxi Province, China, 15 June 1987, Xianjing Peng leg.; 1 ♂ (paratype, HNU), Mt. Huangshan, Anhui Province, China, October 1979, Jiafu Wang leg.

##### Additional material examined.

2 ♂, 7 ♀ (HBU), Tiantangzhai National Forest Park (30°24'01.37"N, 115°18'19.31"E), Hubei, China, 8 September 2012, Fengxiang Liu, Jie Liu and Dan Quan leg.

##### Diagnosis.

**Figures 1–5.**
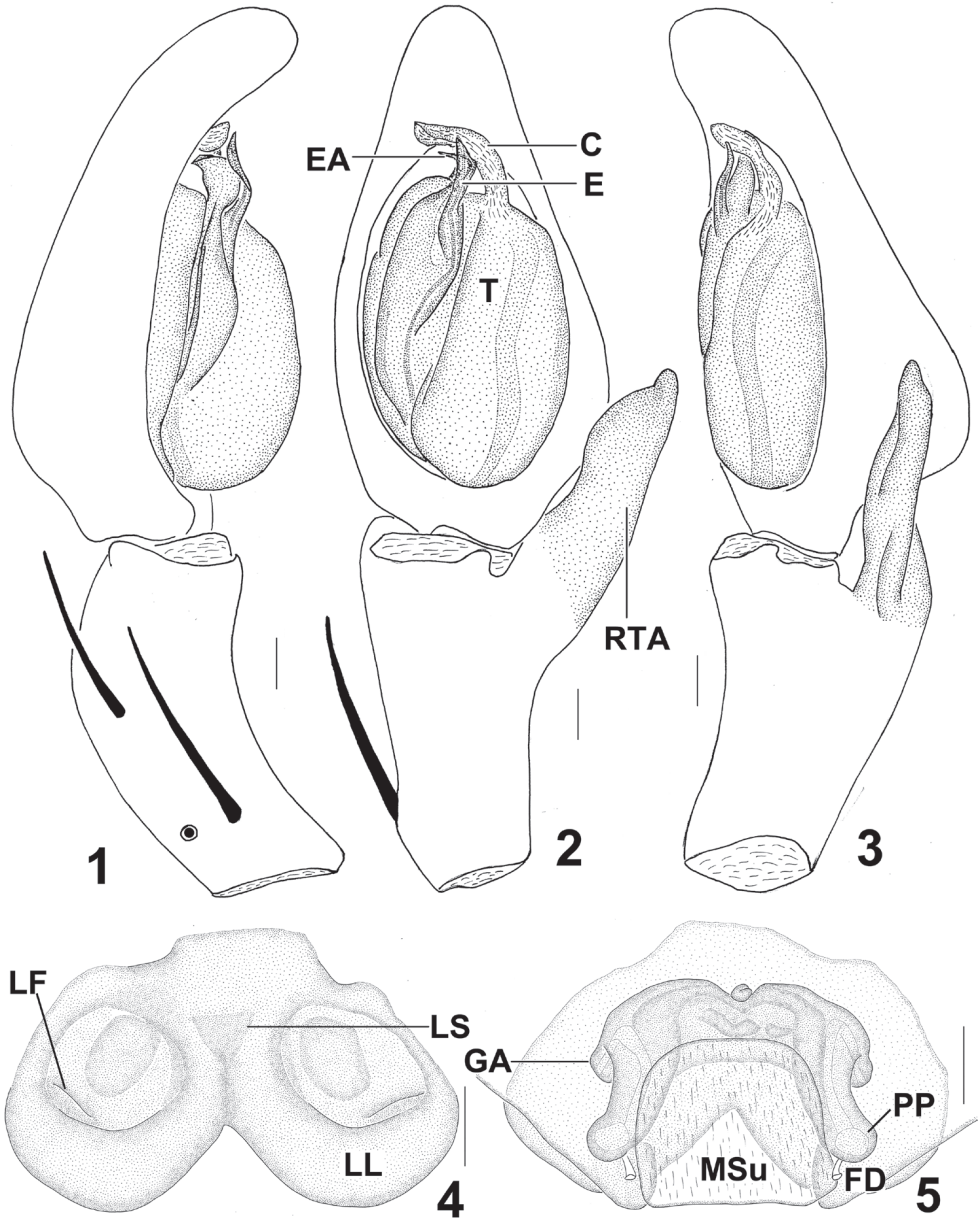
*Sinopoda serrata* (Wang, 1990), from Tiantangzhai National Forest Park (Hubei Province, China). **1** Left male palp, prolateral view **2** Left male palp, ventral view **3** Left male palp, retrolateral view **4** Epigyne, ventral view **5** Vulva, dorsal view. Scales = 0.2 mm. **C** conductor, **E** embolus, **EA** embolic apophysis, **FD** fertilization duct, **GA** glandular appendage, **LF** lateral furrow, **LL** lateral lobes, **LS** lobal septum, **MSu** membranous sac unexpanded, **RTA** retrolateral tibial apophysis, **PP** posterior part of spermathecae, **T** tegulum.

**Figures 6–12.**
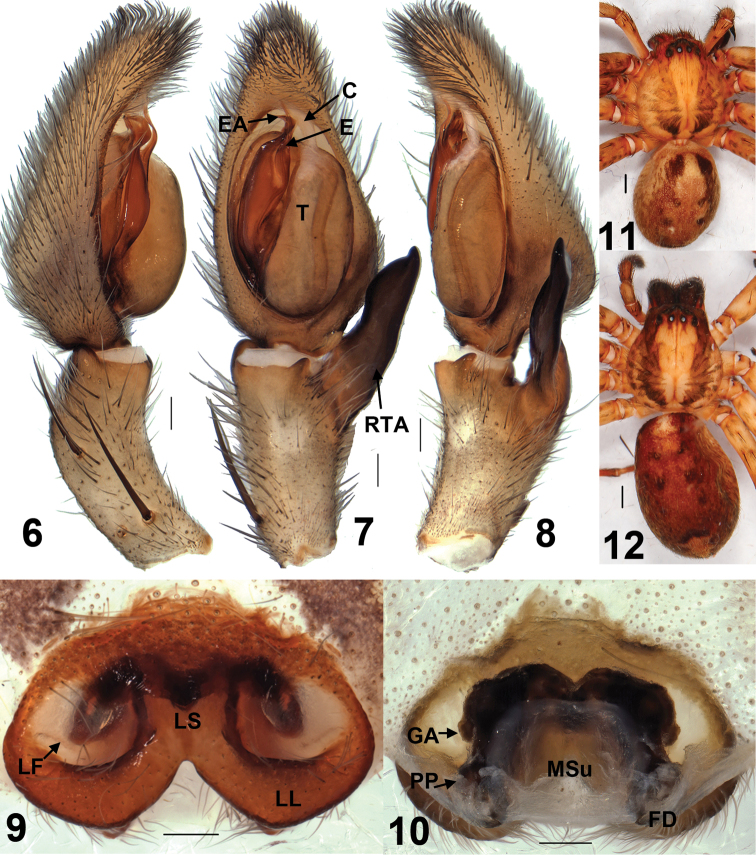
*Sinopoda serrata* (Wang, 1990), from Tiantangzhai National Forest Park (Hubei Province, China). **6** Left male palp, prolateral view **7** Left male palp, ventral view **8** Left male palp, retrolateral view **9** Epigyne, ventral view **10** Vulva, dorsal view**11** Male habitus, dorsal view**12** Female habitus, dorsal view. Scales = 0.2 mm (**6–10**), scales = 1 mm (**11–12**). **C** conductor, **E** embolus, **EA** embolic apophysis, **FD** fertilization duct, **GA** glandular appendage, **LF** lateral furrow, **LL** lateral lobes, **LS** lobal septum, **MSu** membranous sac unexpanded, **RTA** retrolateral tibial apophysis, **PP** posterior part of spermathecae, **T** tegulum.

Male of *Sinopoda serrata* is similar to *Sinopoda albofasciata* Jäger & Ono, 2002 in having the unbifurcated RTA, the slightly bent tip of embolus, but can be distinguished from the latter by the following characters: 1. RTA massive, but small in *Sinopoda albofasciata* (Jäger and Ono 2002); 2. The embolic apophysis (EA) distinctive, lamellar, but reduced in *Sinopoda albofasciata* (Jäger and Ono 2002) (Figs 1–3, 6–8). Female of *Sinopoda serrata* is similar to *Sinopoda undata* Liu, Li & Jäger, 2008 in having the fused lateral lobes (LL), the square-shaped membranous sac unexpanded (MSu), but can be distinguished from the latter by the following characters: 1. Lobal septum (LS) of epigyne significantly short, but long in *Sinopoda undata* (Liu et al. 2008); 2. Internal duct system significantly wider than long, its left part widely separated from right part, but slightly wider than long in *Sinopoda undata*, its left part closed to right part (Liu et al. 2008) (Figs 4–5, 9–10).

##### Description.

**Male:** Measurements: Prosoma length 4.97, width 4.19, anterior width 2.32, height 2.17; opisthosoma length 5.04, width 2.94. Eyes: AME 0.17, ALE 0.33, PME 0.23, PLE 0.29, AME–AME 0.24, AME–ALE 0.10, PME–PME 0.30, PME–PLE 0.37, AME–PME 0.41, ALE–PLE 0.36, clypeus height at AME 0.33, clypeus height at ALE 0.28. Leg and palp measurements: Palp 7.33 (2.36, 1.34, 1.44, -, 2.19), I 20.38 (4.73, 1.97, 6.76, 5.04, 1.88), II 21.99 (5.96, 2.24, 5.61, 6.12, 2.06), III 16.22 (4.73, 1.63, 4.16, 4.10, 1.60), IV 18.16 (5.01, 1.73, 4.40, 5.03, 1.99). Leg formula: II-I-IV-III. Spination: palp 131, 101, 1021; femur I–III 323, IV 321; patella I–IV 101; tibia I–II, III 2126, IV 2326; metatarsus I–II 1014, III–IV 3036. Chelicerae yellowish-brown. Furrow with 3 anterior teeth, 4 or 6 posterior teeth, and with ca.80 denticles in elongated patch close to anterior teeth. Margins of fang base with one bristle. Palpal claw with 6 or 7 teeth. Sternum, ventral coxae and femora, distal legs as well as frontal chelicerae with long setae, otherwise with shorter setae.

Embolus (E) tip short, slender, slightly curved prolaterally, proximal part of embolus fully visible in the ventral view. Embolic apophysis (EA) short, lamellar, strongly curved prolaterally. Sperm duct (SD) curved in ventral view. RTA large, not bifurcate, arising distally from tibia. Cymbium slightly longer than tibia (Figs 1–3, 6–8).

Colouration in ethanol (Fig. 11): Yellowish- to slightly yellowish-brown. Dorsal prosoma yellowish-brown with petaline patterns, which are divided by the bright yellowish region between posterior eye row and posterior margin of carapace. Sternum, ventral coxae and femora, gnathocoxae, and labium pale yellowish-brown, gnathocoxae and labium proximally reddish-brown. Chelicerae yellowish-brown. Legs pale yellowish-brown with distal parts slightly darker, dorsal femora with dark pattern. Dorsal opisthosoma with two pairs of spots situated in the median part, with a pale yellow inverted triangle-shaped pattern near the spinnerets. Lateral parts of opisthosoma reddish-brown, Ventral opisthosoma with little dark patterns.

**Female:** Measurements: Prosoma length 4.94, width 4.52, anterior width 2.49, height 2.15; opisthosoma length 7.33, width 4.86. Eyes: AME 0.22, ALE 0.31, PME 0.22, PLE 0.32, AME–AME 0.22, AME–ALE 0.11, PME–PME 0.32, PME–PLE 0.52, AME–PME 0.43, ALE–PLE 0.40, clypeus height at AME 0.32, clypeus height at ALE 0.33. Leg and palp measurements: Palp 6.23 (1.98, 0.94, 1.34, -, 1.97), I 14.37 (4.13, 1.82, 3.80, 3.51, 1.47), II 16.42 (4.61, 2.18, 4.26, 3.91, 1.46), III 13.42 (4.05, 1.68, 3.24, 3.15, 1.30), IV 15.64 (4.46, 1.54, 3.72, 4.16, 1.76). Leg formula: II-IV-I-III. Spination: palp 131, 100, 2121, 1014; femur I–III 323, IV 321; patella I 101, II–IV 001; tibia I–III 2026, IV 2326; metatarsus I–II 1014, III–IV 3036. Chelicerae yellowish-brown. Furrow with 3 anterior teeth, 4 posterior teeth, and with ca.80 denticles in elongated patch close to anterior teeth. Margins of fang base with one bristle. Palpal claw with 7 teeth. Sternum, ventral coxae and femora, distal legs as well as frontal chelicerae with long setae, otherwise with shorter setae.

Epigynal field wider than long. Lateral lobes (LL) fused, posteriorly with median incision. Epigynal pockets running from latero-posterior to medio-anterior, where copulatory openings are situated. Lateral furrows (LF) distinct, running to lateral margins of lateral lobes. Lobal septum (LS) wide, significantly short. Internal duct system significantly wider than long, its left part widely separated from right part. Glandular appendages (GA) small, extending not in posterior half of internal duct system. Posterior part of spermathecae (PP) strongly short, bulging slightly laterally. Fertilization ducts (FD) arising posterio-laterally. Membranous sac between fertilisation ducts unexpanded, almost square-shaped (Figs 4–5, 9–10).

Colouration in ethanol (Fig. 12) as in male.

##### Remarks.

There is a small difference between the holotype male and the new collected materials: the middle part of RTA slightly covered the cymbium from the ventral view in the new collected materials, but not in the holotype (Figs 2, 7, 14).

**Figures 13–16.**
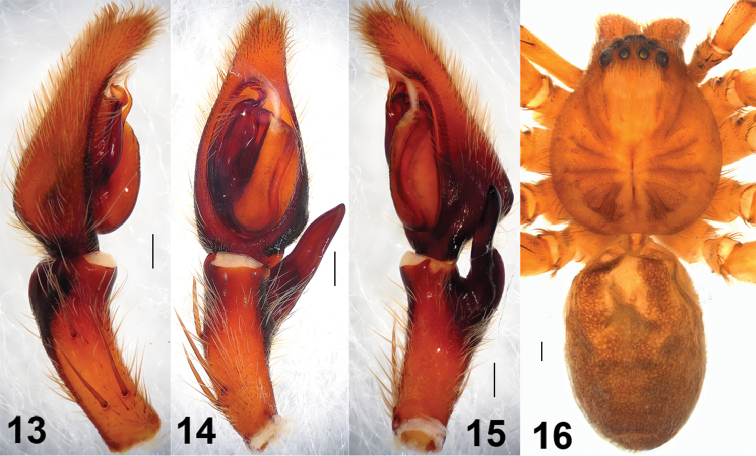
*Sinopoda serrata* (Wang, 1990), holotype, from Mt. Lushan (Jiangxi Province, China). **13** Left male palp, prolateral view **14** Left male palp, ventral view **15** Left male palp, retrolateral view **16** Male habitus, dorsal view. Scales = 0.2 mm (**13–15**), scale = 1 mm (**16**).

##### Distribution.

**Figure 17.**
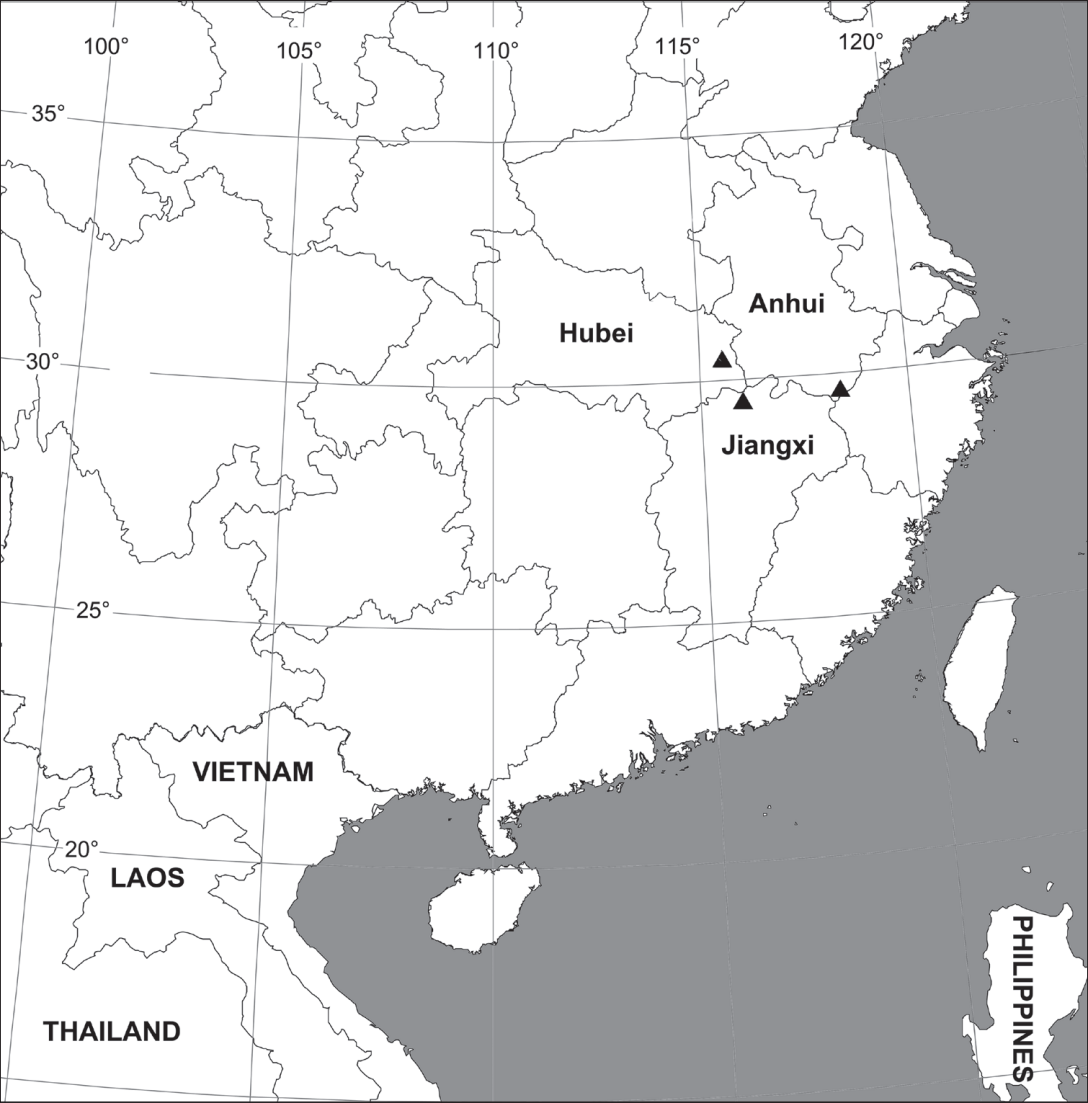
Collection localities of *Sinopoda serrata* (Wang, 1990) in China.

China (Hubei, Jiangxi, Anhui) (Fig. 17).

## Supplementary Material

XML Treatment for Sinopoda serrata
